# Preparation of the inactivated Newcastle disease vaccine by plasma activated water and evaluation of its protection efficacy

**DOI:** 10.1007/s00253-019-10106-8

**Published:** 2019-11-16

**Authors:** Zhou Hongzhuan, Tian Ying, Su Xia, Guo Jinsong, Zhang Zhenhua, Jiang Beiyu, Chang Yanyan, Lin Lulu, Zhang Jue, Yang Bing, Fang Jing

**Affiliations:** 1 Beijing Key Laboratory for Prevention and Control of Infectious Diseases in Livestock and Poultry, Institute of Animal Husbandry and Veterinary Medicine, Beijing Academy of Agriculture and Forestry Sciences, Beijing, 100097 People’s Republic of China; 2 Academy for Advanced Interdisciplinary Studies, Peking University, Beijing, 100871 People’s Republic of China; 3 College of Engineering, Peking University, Beijing, 100871 People’s Republic of China

**Keywords:** Plasma activated water, Newcastle disease virus, Inactivated vaccine preparation, Immune response

## Abstract

Vaccination has been regarded as the most effective way to reduce death and morbidity caused by infectious diseases in the livestock industry. In this study, plasma activated water (PAW) was introduced to prepare the inactivated Newcastle disease vaccine. Humoral immune response was tested by hemagglutination inhibition (HI) assay and enzyme-linked immunosorbent assay (ELISA). In addition, cell-mediated immune response was evaluated by lymphocyte proliferation assay and flow cytometry. The results demonstrated that the vaccine prepared by PAW at appropriate volume ratio could induce similar antibody titers in specific pathogen-free (SPF) chickens compared with the formaldehyde-inactivated vaccine. The challenge experiment further confirmed that the vaccine prepared by PAW conferred solid protection against virulent NDV. Moreover, it was found that the vaccine could promote the proliferation of lymphocytes and stimulate cell-mediated immunity of SPF chickens. Furthermore, analysis of electron spin resonance (ESR) spectroscopy and physicochemical properties of PAW suggested reactive oxygen and nitrogen species (RONS) played an essential role in the virus inactivation. Therefore, this study indicated that NDV treated by PAW in an appropriate ratio retained immunogenicity on the premise of virus inactivation. PAW as a promising strategy could be used to prepare inactivated vaccine for Newcastle disease.

**Electronic supplementary material:**

The online version of this article (10.1007/s00253-019-10106-8) contains supplementary material, which is available to authorized users.

## Introduction

Newcastle disease (ND) is a worldwide infectious disease in the livestock industry, which can infect plenty of wild and domestic avian species; also, ND is endemic in many developing parts of the world and spreads easily via different routes (Alexander 2000a; Ganar et al. 2014; Seal et al. 2000). It has been recognized as a notifiable disease in view of its rapid transmission and a large scale of impact in poultry industry (Alexander et al. 2012). Outbreaks of ND may be destructive and the mortality of Velogenic ND was approximately 100% in poultry (Alexander 2000b; Ganar et al. 2014). Virulent NDV infection is usually accompanied by expiratory dyspnea, depression, hemorrhage in multiple organs, and acute death. Therefore, NDV infection has been responsible for severe economic losses in the livestock industry, which needs to attract attention (Alexander 2001; Ganar et al. 2014; Wise et al. 2004). In order to control and prevent the spread of ND, the current common practice worldwide is vaccination (Alexander 2001).

The licensed antiviral vaccines mainly divide into three broad categories: conventional inactivated vaccine, live attenuated vaccines, and gene-manipulated vaccines (Wang et al. 2016). The conventional inactivated vaccines usually depend on formaldehyde and β-propiolactone (BPL), which are the most common inactivated reagents. Numerous studies have reported that formaldehyde may have adverse effects on human health or even cause more severe disease under certain conditions. For example, vaccination with formaldehyde-inactivated respiratory syncytial virus (RSV) vaccine may not be protective and may result in more serious RSV infection (Brown 1993; Muralidharan et al. 2017). BPL may induce the allergic reactions caused by chemical modifications (Stauffer et al. 2006; Swanson et al. 1987). With regard to commercial gene-manipulated vaccines, the safety and efficacy remains to be fully evaluated before they are put on the market (Delany et al. 2014; Ulmer et al. 2006). In view of the above reasons of current vaccines, there is a need for an alternative technique that is safer and lower in cost for preparing inactivated vaccine.

The non-thermal plasma technology has a promising prospect in various biomedical fields, such as environmental pollution (Chen et al. 2010; Zhang et al. 2016), food preservation (Baier et al. 2013; Ma et al. 2015; Pankaj et al. 2014; Thirumdas et al. 2015), and inhibition of cancer cell growth (Kim et al. 2010a; Liedtke et al. 2017). It has been realized that reactive oxygen and nitrogen species (RONS) produced by non-thermal plasma are the major bactericidal agents, which can lead to oxidative stress in microbial cells, consequently resulting in damage to nucleic acid and proteins (Kim et al. 2010b). Wang et al. first reported that the non-thermal plasma could be used to formulate the inactivated vaccine, which established contacts between the physical technology and poultry vaccines (Wang et al. 2016).

Plasma activated water (PAW), which is obtained by non-thermal plasma activation, also has the potential in biomedical fields including the disinfection of medical instrument (Pan et al. 2017), fruit fresh-keeping, and tooth whitening (Ma et al. 2016; Xu et al. 2016). Generally, non-thermal plasma is an ionized gas consisting of charged particles, ultraviolet rays, and electrons in addition to RONS (Deng et al. 2006), which are more detrimental to the biological surfaces. Therefore, it is a challenge to control non-thermal plasma energy and avoid the damage of key ingredient (Kong et al. 2009; Morfill et al. 2009). Meanwhile, PAW as antiviral solution is environmentally friendly, which has an advantage over the traditional chemical sanitizers (Wei et al. 1985). Considering its easy to control and environmentally friendly features, we try to adopt PAW application. It is noteworthy that Su et al. have proposed that PAW possessed the potential ability of virus inactivation (Su et al. 2018), which lay a foundation for evaluating the PAW ability for vaccine preparation and its protective efficacy.

The objective of this study was to investigate the feasibility of PAW on inactivated vaccine preparation and assess the immune response of PAW-inactivated vaccine. The antiviral ability of PAW was assessed by embryo lethality assay (ELA) and hemagglutination (HA) test. Afterwards, the NDV-specific antibodies of specific pathogen-free (SPF) chickens were detected by HI assay and enzyme-linked immunosorbent assay (ELISA). In addition, the immune responses of SPF chickens post-vaccination were tested by the lymphocyte proliferation assay and flow cytometry analysis. Furthermore, the physicochemical properties of PAW and virus solution were measured including oxidation-reduction potential (ORP), pH values, and NO radical concentration.

## Methods and materials

### Plasma microjet device and PAW generation

The air plasma generator was described in detail in previous reports (Yu et al. 2015). The device was designed based on dielectric barrier structure with hollow electrodes (HEDBS) structure, which consisted of copper electrode and quartz dielectric. When air with 260 L/h was forced into the quartz tube, a homogeneous plasma was generated in the discharge region and ejected through the outlet end.

**Fig. 1:**
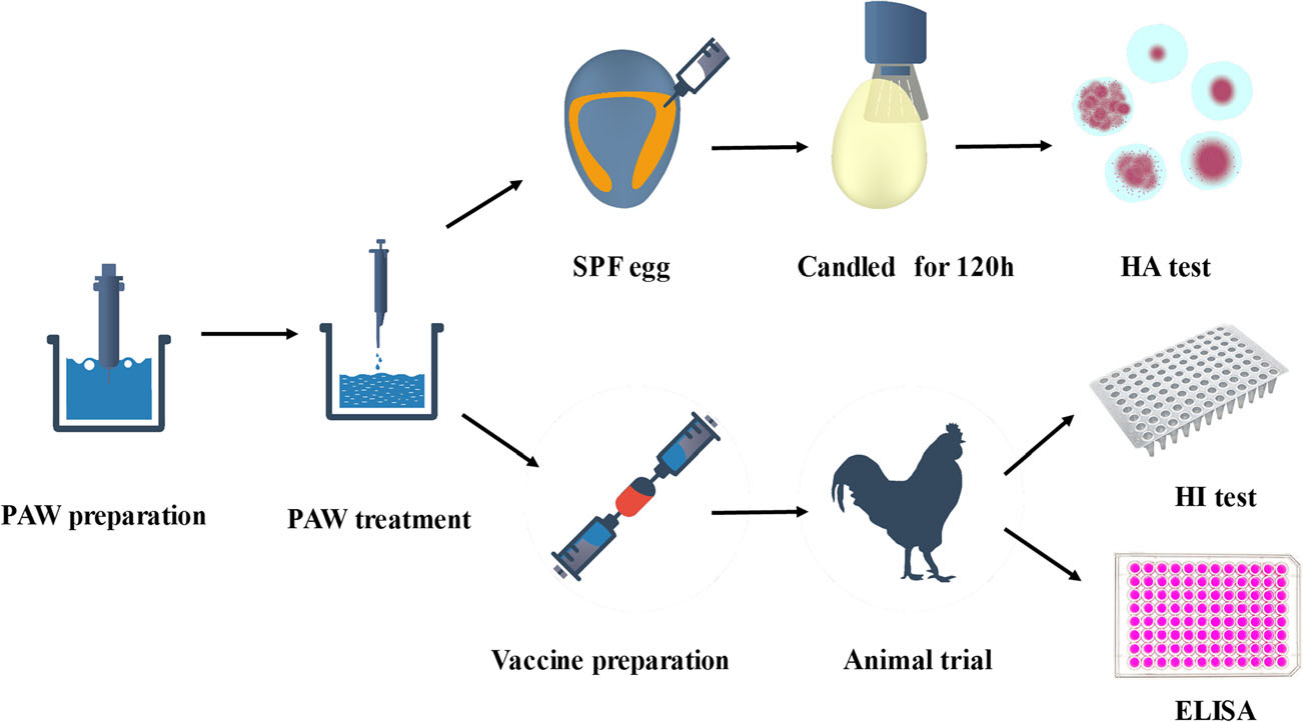
Experimental procedure description including PAW treatment, virus inactivation, and vaccination

As presented in Fig. 1, PAW was obtained by placing the plasma microjet (PMJ) beneath the water surface. The distance between the outlet end of plasma jet and water surface was approximately 20 mm. Each of 20 mL sterile distilled water was activated by non-thermal plasma for 30 min to obtain PAW, which were freshly used for subsequent experiments.

### Virus proliferation and titer detection

The NDV LaSota strain, obtained from the China Veterinary Culture Collection Center (CVCC), was propagated in the allantoic cavity of 10-day-old SPF chicken embryos (Beijing Merial Vital Laboratory Animal Technology Co., Ltd., Beijing, China) at 37 °C. The allantoic fluid-containing virus was collected from the chicken embryos, then centrifuged at 1000×*g* and stored at − 20 °C before use. The virus titer was 10^8.5^ egg infectious dose (EID_50_)/0.1 mL, which were calculated by the method of Reed and Muench after serial titration in eggs (Reed and Muench 1938; Zitzow et al. 2002).

### PAW treatment

To verify the inactivation ability, 9 mL, 5 mL, and 2 mL PAW solution has been mixed with 1 mL allantoic fluids containing virus for 2 h, respectively. For a simplified description, they were defined as group A, group B, and group C, respectively. NDV-allantoic fluid without PAW treatment was regarded as the control. The ELA and HA test were employed to determine the virus inactivation.

### Embryo lethality assay

Viruses treated by PAW at different volume ratios and the control sample were inoculated into the 10-day-old SPF chicken embryos at 37 °C. Each group had five chicken embryos. Based on the previous reports (King 1999; Westbury 1979), the chicken embryos were candled every 24 h and observed for 120 h. The death number beyond 24 h was recorded as the ELA results.

### Hemagglutination test

HA tests were conducted by conventional microtiter plates (Westbury 1979). Fifty microliters of 1% chicken erythrocytes was added to equal volume of serial twofold dilutions of samples, which were diluted with saline solution. After slight oscillation, the plates were incubated at room temperature for 25 min. It was considered positive that wells contained a homogeneous and adherent layer of erythrocytes. The HA titer was recorded as log_2_ of the highest dilution of antigen giving complete HA.

### Immunization

The SPF chickens were raised in negative pressure isolators (Suzhou Fengshi Laboratory Animal Equipment Co., Ltd., GJ-2, China) during the experiments. The SPF chickens were provided by Beijing Merial Vital Laboratory Animal Technology Co., Ltd., China. The 28-day-old SPF chickens were labeled and randomly assigned into five groups (*n* = 20/group), including saline, formaldehyde, group A, group B, and group C. The oil adjuvant was mixed uniformly with the PAW-treated NDV antigens and formaldehyde-treated NDV antigens to prepare inactivated oil-emulsified vaccines (Ong et al. 2010; Thim et al. 2012). The saline group denoted that the SPF chickens were injected saline solution only. The chickens were vaccinated by intramuscular injection with prepared vaccine containing 10^8.3^ EID_50_ NDV antigen correspondingly. Chicken sera were obtained via the wing vein 14, and 21 days post-immunization. Sera were separated after centrifugation at 8000 rpm and stored at − 20 °C until use. The antibody titers in sera samples were determined via HI and ELISA.

Three weeks after immunization, all the chickens were challenged with 0.5 mL of 10^5^ ELD_50_ velogenic NDV by intramuscular injection (Zimmermann et al. 2011). The chickens were monitored daily after challenge and the death numbers of chickens were recorded. The dead chickens were stored at 4 °C. All live chickens were killed by intravenous pentobarbital sodium (Merck, Germany) after 10 days (Wang et al. 2016).

### Hemagglutination inhibition test

Serial twofold serum dilutions were done in saline solution, which were mixed with an equal volume of 4HA units NDV antigen (25 μL). After incubation for 25 min, 25 μL of 1% chicken erythrocytes was added to the mixture. The HI titer was expressed as log_2_ of the reciprocal of the highest dilution giving complete inhibition of HA.

### NDV-specific antibody titer by enzyme-linked immunosorbent assay

The sera harvested after 14 and 21 days post-immunization were tested by a commercial ELISA kit (IDEXX Laboratories Inc., Westbrook, ME) for evaluation of NDV specific-antibodies. The sera samples were at a dilution of 1:500 and incubated in 96-well plates containing virus antigen. The experiment was carried out in accordance with the instruction of manufacturers. NDV specific-antibody titers in the sera samples were analyzed according to the presented method (Kapczynski and King 2005; Loke et al. 2005). The presence or absence of antibody to NDV is determined by relating the absorbance value at 650 nm of the sample to the positive control mean. The positive control is standardized and represents significant antibody levels to NDV in chicken serum. The relative level of antibody in the sample is determined by calculating the sample to positive (S/P) ratio. Based on the information provided by the instruction, the S/P ratio as an indicator of antibody titer was considered positive when greater than 0.2.

### Lymphocyte proliferation

The peripheral blood samples were collected from the wing vein 14 and 21 days post-vaccination, which were immediately added into the anticoagulant tubes. The peripheral blood lymphocytes (PBLs) were isolated by a commercial chicken peripheral blood lymphocytes extraction kit (Solarbio, P8740) and resuspended in RPMI1640 complete medium with 10% fetal calf serum and 100 IU/mL penicillin, and 100 μg/mL streptomycin. The trypan blue dye was used to determine the cell counts, which was adjusted to 1 × 10^7^ cells/mL. The cells (100 μL) were seeded in 96-well cell culture plates and were treated with 10 μg/mL concanavalin A (ConA). The plates were incubated in the culture chamber at 37 °C with 5% CO_2_ for 68 h. Finally, 10 μL of CCK-8 (Sigma, St. Louis, MO) was added and incubated for 4 h at the same condition. The optical density (OD) readings were measured at 450 nm on a SPECTROstar Omega absorbance plate reader with a Rapid UV/Vis spectrometer (BMG, Germany).

### Flow cytometry

Cell-mediated immune response is vital for clearing intracellular pathogens, and is an important measurable indicator of vaccine’s effectiveness. The PBLs were collected for the detection of total CD3^+^ T cells and their subsets (CD4^+^ T cells [CD3^+^CD4^+^] and CD8^+^ T cells [CD3^+^CD8^+^]), which were isolated as previously described. Subsequently, 1 × 10^6^ lymphocytes were incubated with corresponding antibodies including anti-chicken CD3-SPRD, anti-chicken CD4-FITC, and anti-chicken CD8-PE (Southern Biotech, USA) at 4 °C for 40 min. Then, the lymphocytes were washed with phosphate-buffered saline (PBS) and were resuspended in a volume of 300 μL. An aliquot of 1 × 10^4^ cells per sample was analyzed for positive staining by FACSDiva sofware (Becton, Dickinson, USA) (Hiremath et al. 2016).

### Detection of physicochemical properties

ORP was used to characterize the general oxidation ability of solution (McFerson 1993). The ORP values of group A, group B, and group C were performed by using an ORP probe (LE501&510, Mettler Toledo, USA) following the operation procedure. The experiment was randomly carried out three times. The corresponding pH was recorded immediately after the treatment with a pH-meter (Mettler-Toledo LE438).

Electron spin resonance spectroscopy (ESR) was employed to detect the short-lived reactive species in group A, group B, and group C. The pivotal nitric oxide radical (NO•) in samples was trapped by adding 250 μL N-methyl-D-glucamine dithiocarbamate MGD (1.0 M) (99%, J&K Scientific Ltd. China) and 250 μL Fe^2+^ (0.3 M), ultimately forming the longer-lived spin adduct complex NO-Fe^2+^(MGD)_2_ (Halliwell 2006; Palmer et al. 1987). The final substrate was imbibed by a capillary and tested in the resonator cavity of ESR spectrometer (ER-200D-SRC/E-500, Bruker Ltd., German) operated at room temperature.

### Statistics analysis

Data were obtained from at least three replicate experiments (*n* ≥ 3). Results were presented as the mean ± standard deviation (SD). Statistical analysis was carried out using SPSS statistical package 17.0 (SPSS Inc., USA). An analysis of variance (ANOVA) was performed to compare the antibody titers and immune responses induced by different groups, as well as the ORP and pH values of different groups. Significant differences between mean values were identified by the Duncan method with a confidence level at *P* ≤ 0.05. In addition, the paired-sample *t* test was employed to compare the inactivation efficacy of PAW at different conditions against NDV.

## Results

### Virus inactivation by PAW

To investigate the feasibility of PAW on preparing inactivated vaccine, it was first verified that the virus was inactivated by PAW completely. As shown in Fig. S1(a), the survival rates of chicken embryos were all 100% after injection with PAW-treated NDV for 120 h. As for the control group, the survival rate decreased to 80% after 72 h incubation, and the embryos were all dead at 120 h. Subsequently, the allantoic fluids were collected from embryos inoculated with PAW-treated NDV. The HA titers in PAW groups were not detectable as compared with 8.3log_2_ of the control group (Fig. S1(b)). In combination with ELA and HA results, it was found that PAW could inactivate NDV completely.

### Antibody titer induced by PAW-inactivated vaccine

**Fig. 2:**
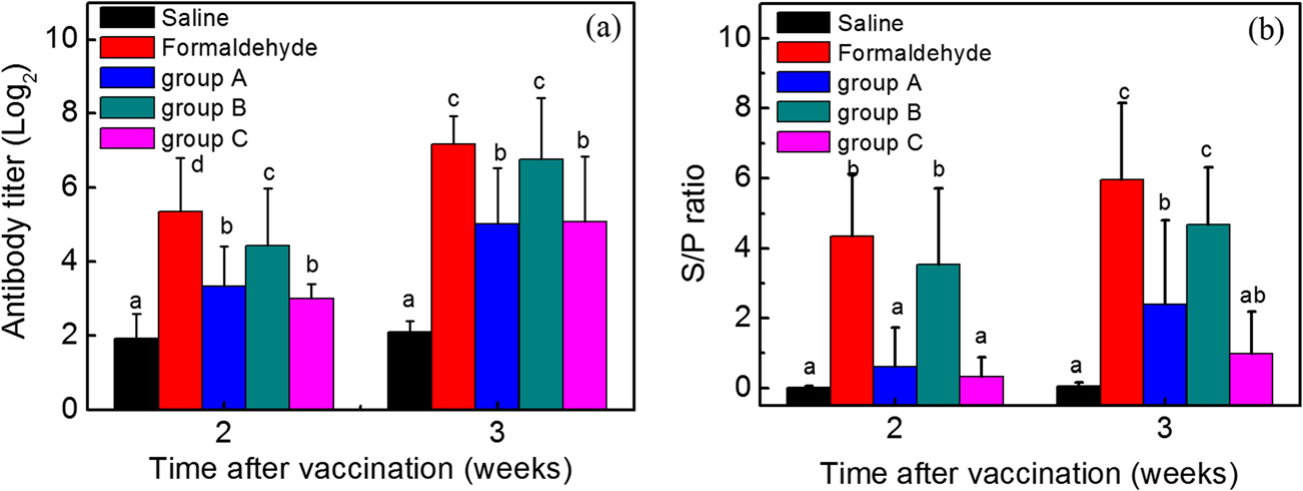
Antibody titers of vaccinated chickens. (a) The influence of PAW on antibody titers determined by HI. (b) The influence of PAW on antibody titers determined by ELISA. Bars labeled with different letters (a–d) across different treatment groups represent a significant difference at the same day post-vaccination (*P* ≤ 0.05). Saline, chicken injected with saline; Formaldehyde, chicken injected with inactivated vaccine prepared by formaldehyde; group A, chicken injected with inactivated vaccine prepared by PAW and PAW interacted with virus suspension at a volume ratio of 9:1 (PAW: virus = 9:1); group B, chicken injected with inactivated vaccine prepared by PAW and PAW interacted with virus suspension at a volume ratio of 4:1 (PAW: virus = 4:1); group C, chicken injected with inactivated vaccine prepared by PAW and PAW interacted with virus suspension at a volume ratio of 2:1 (PAW: virus = 2:1). An analysis of variance (ANOVA) was performed to compare the antibody titers induced by different groups. Significant differences between mean values were identified by the Duncan method with a confidence level at *P* ≤ 0.05

After vaccination, NDV-specific antibody titers of the sera harvested from vaccinated chickens in all groups were assessed. As presented in Fig. 2(a), 2 weeks after vaccination, the antibody levels of group A, group B, and group C were 3.33 ± 1.08log_2_, 4.44 ± 1.55log_2_, and 3.00 ± 0.39log_2_ titers. In contrast, the antibody levels of the saline group were only 1.92 ± 0.67log_2_. NDV-specific antibody levels of SPF chickens vaccinated with group A and group C were comparable. As for the formaldehyde group, the antibody showed higher titers than that of other groups, reaching 5.36 ± 1.45log_2_ (*P* < 0.05). Three weeks after vaccination, chickens injected with inactivated vaccine prepared by formaldehyde and group B appeared to be a similarly increased trend, achieving 7.18 ± 0.75log_2_ and 6.77 ± 1.64log_2_, respectively, which have no significant difference. The antibody level of the saline group had almost no change, maintaining at 2.09 ± 0.30log_2_. High doses of group A and low doses of group C stimulated the production of the antibody titers, which were between the saline group and formaldehyde group.

In order to further confirm the effectiveness of PAW-inactivated vaccine, serum was also analyzed for anti-virus antibody content using a commercial ELISA kit. As presented in Fig. 2(b), anti-NDV antibodies in vaccinated chickens were detected 2 weeks post-immunization. The S/P ratios observed in vaccinated chickens of the formaldehyde group and group B were comparable after 2 weeks, displaying 4.34 ± 1.77 and 3.53 ± 2.18, respectively, which were significantly higher than those in group A (0.62 ± 1.10) and group C (0.34 ± 0.54). Three weeks post-vaccination, the S/P ratios of group B increased to 4.67 ± 1.65. By contrast, the antibody titers chickens injected with group A and group C were observed lower S/P ratios (2.40 ± 2.40 and 0.99 ± 1.20). The S/P ratio of the saline group remained low, about 0.04 ± 0.03. All data were in good agreement with the results of the HI test.

### Lymphocyte proliferation

**Fig. 3:**
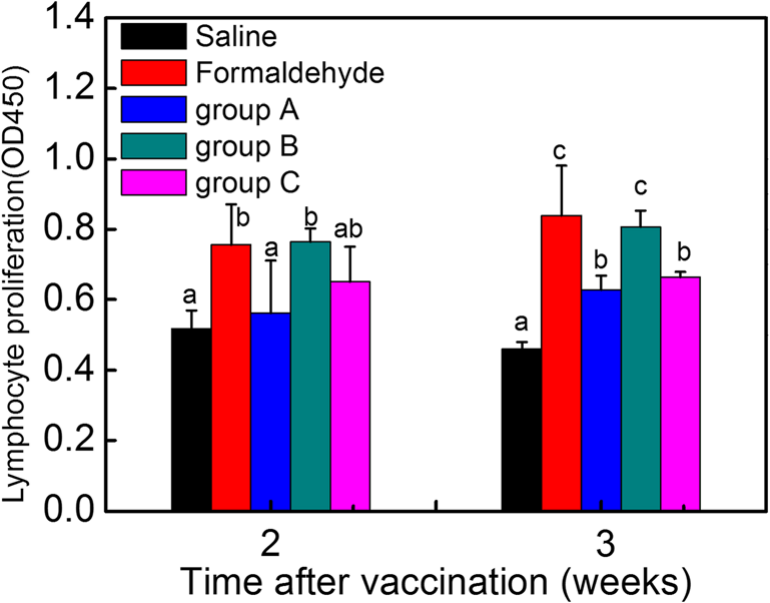
Results of the lymphocyte proliferation after vaccination. An analysis of variance (ANOVA) was performed to compare immune responses induced by different groups. Significant differences between mean values were identified by the Duncan method with a confidence level at *P* ≤ 0.05. Bars labeled with different letters (a–d) across different treatment groups represent a significant difference 2 and 3 weeks post-vaccination (*P* ≤ 0.05). Saline, chicken injected with saline; Formaldehyde, chicken injected with inactivated vaccine prepared by formaldehyde; group A, chicken injected with inactivated vaccine prepared by PAW and PAW interacted with virus suspension at a volume ratio of 9:1 (PAW: virus = 9:1); group B, chicken injected with inactivated vaccine prepared by PAW and PAW interacted with virus suspension at a volume ratio of 4:1 (PAW: virus = 4:1); group C, chicken injected with inactivated vaccine prepared by PAW and PAW interacted with virus suspension at a volume ratio of 2:1 (PAW: virus = 2:1)

The lymphocyte proliferation experiment was performed to evaluate the effect of inactivated vaccine formulated by PAW on cellular immunity. As shown in Fig. 3, 2 weeks post-vaccination, the lymphocyte proliferation response of SPF chickens immunized with group B and formaldehyde-inactivated vaccine were enhanced compared with those immunized with saline, reaching 0.76 ± 0.12 and 0.76 ± 0.04, respectively. Comparably, the lymphocyte proliferation of group A and group C was as low as that of the saline group (*P* > 0.05). Three weeks after vaccination, compared with the saline group, although the lymphocyte proliferation response of chickens immunized with group A and group C increased slightly, achieving 0.63 ± 0.04 and 0.66 ± 0.02, accordingly, the three groups were still at a lower level. Meanwhile, group B stimulated similar lymphocyte proliferation response with regard to the formaldehyde-inactivated vaccine, which was higher than other groups. These data indicated that PAW-inactivated vaccine, especially group B could promote lymphocyte proliferation.

### Immunophenotyping of T lymphocyte subsets

Chicken peripheral blood lymphocytes were separated, and the percentages of T cell subsets were monitored by flow cytometry. As shown in Table 1, 2 weeks after vaccination, the percentages of total CD3^+^ T cells in the peripheral blood lymphocyte populations of chickens immunized with group B were significantly higher than that in the chickens immunized with group A and group C. In addition, group B showed greater potential and produced more CD4^+^ and CD8^+^ T cells, about 27.2 ± 1.27% and 9.3 ± 2.28%, compared with the saline group. As for the formaldehyde group, the T cell subsets show higher percentages than groups A and C. Meanwhile, there was no significant difference between the formaldehyde group and group B.

**Table 1:** Flow cytometric analysis of CD3^+^ T cells and CD3^+^CD4^+^ and CD3^+^CD8^+^ T cell subsets from peripheral blood lymphocytes of immunized chickens

Treatment	% of peripheral blood lymphocytes in T cells						
	14 d.p.i				21 d.p.i		
	CD3^+^	CD3^+^CD4^+^		CD3^+^CD8^+^	CD3^+^	CD3^+^CD4^+^	CD3^+^CD8^+^
Saline	22.1 ± 7.28^ab^	13.9 ± 4.08^a^		4.1 ± 0.92^a^	34.5 ± 2.05^a^	16.1 ± 0.54^a^	12.5 ± 2.18^a^
Formaldehyde	40.5 ± 4.73^cd^	26.6 ± 2.50^b^		7.4 ± 0.96^ab^	40.3 ± 12.19^ab^	25.5 ± 2.62^cd^	10.7 ± 6.20^a^
Group A	29.3 ± 8.67^bc^	15.1 ± 6.15^a^		9.4 ± 3.82^b^	43.1 ± 3.85^ab^	20.1 ± 1.75^ab^	9.4 ± 2.92^a^
Group B	45.6 ± 7.37^d^	27.2 ± 1.27^b^		9.3 ± 2.28^b^	48.5 ± 6.70^b^	26.7 ± 4.27^d^	11.0 ± 0.72^a^
Group C	16.1 ± 1.63^a^	8.3 ± 0.49^a^		3.6 ± 1.23^a^	40.3 ± 3.98^ab^	21.3 ± 3.12^bc^	8.8 ± 0.38^a^

An analysis of variance (ANOVA) was performed to compare T cell subsets induced by different groups. Significant differences between mean values were identified by the Duncan method with a confidence level at *P* ≤ 0.05. Different small letters in the same column indicate statistically significant differences (*P* < 0.05)

Three weeks post-vaccination, the increases of CD3^+^ cells were observed in all immunized groups except the formaldehyde group, and CD3^+^ cells in the chickens immunized with group A (43.1 ± 3.85%) and group B (48.5 ± 6.70%) were higher than that with the formaldehyde group (40.3 ± 12.19%), while lower CD3^+^ cells were observed in the chickens immunized with the saline group (34.5 ± 2.05%). Moreover, chickens immunized with group B were exhibited to have a higher percentage of CD3^+^CD4^+^ cells compared with group A and group C, which was 26.7 ± 4.27%. As for the percentage of CD8^+^ cells, no significant differences were detected between PAW treatment groups and the saline group. These data suggested that PAW could stimulate both CD3^+^ and CD4^+^ T cells. Nevertheless, the effect of PAW on CD8^+^T cell percentage was not obvious.

### Protection efficacy of PAW-vaccine against NDV challenge

**Fig. 4:**
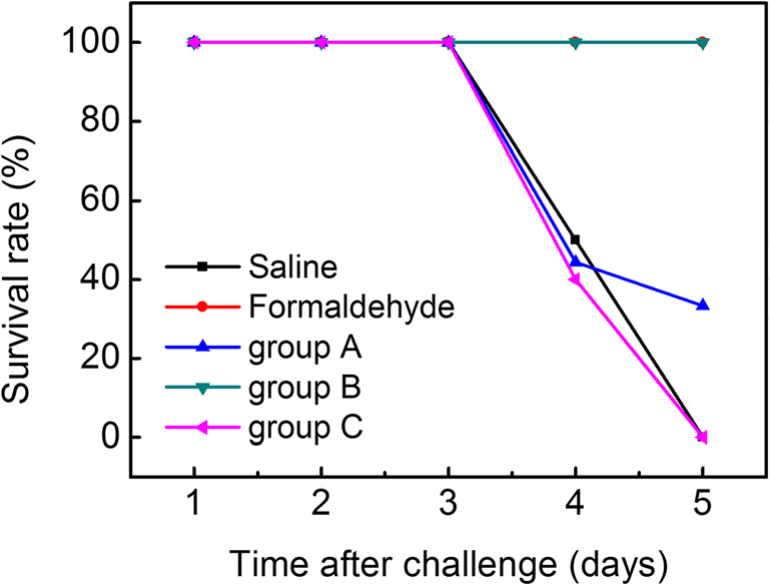
Survival rates of vaccinated chickens against NDV challenge. Saline, chicken injected with saline; Formaldehyde, chicken injected with inactivated vaccine prepared by formaldehyde; group A, chicken injected with inactivated vaccine prepared by PAW and PAW interacted with virus suspension at a volume ratio of 9:1 (PAW: virus = 9:1); group B, chicken injected with inactivated vaccine prepared by PAW and PAW interacted with virus suspension at a volume ratio of 4:1 (PAW: virus = 4:1); group C, chicken injected with inactivated vaccine prepared by PAW and PAW interacted with virus suspension at a volume ratio of 2:1 (PAW: virus = 2:1)

To verify whether the humoral responses induced by PAW-inactivated vaccine provided overall protection, all groups of chickens were challenged with velogenic NDV 3 weeks post-vaccination. No evident clinical symptoms of Newcastle disease appeared in any group before challenge. As shown in Fig. 4, all chickens immunized with formaldehyde-inactivated vaccine and group B survived, conferring 100% protection. However, the chickens that received saline and group C showed serious clinical signs of ND and died within 5 days after NDV challenge. Low level of protection was obtained for group A and only six out of twenty chickens survived. Thus, group B demonstrated excellent protective efficacy in chickens.

### Physicochemical properties

**Fig. 5:**
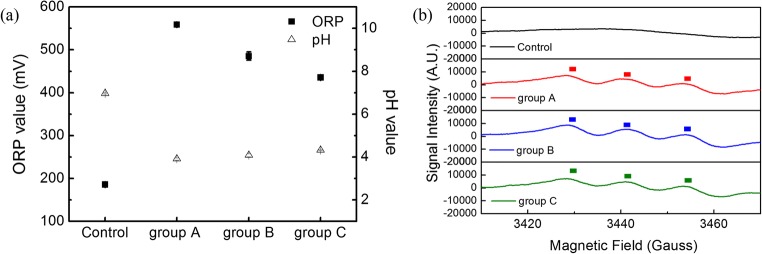
(a) Results of pH and ORP values under PAW treatments. pH, open triangular symbol; ORP, filled square symbol. An analysis of variance (ANOVA) was performed to compare the ORP and pH values of different groups. Significant differences between mean values were identified by the Duncan method with a confidence level at *P* ≤ 0.05. Different small letters indicate statistically significant differences among ORP values of different treatment groups (*P* < 0.05). Different capital letters indicate statistically significant differences among pH values of different treatment groups (*P* < 0.05). (b) Electron spin resonance spin-trapping spectra of NO-Fe^2+^(MGD)_2_ in control, group A, group B, and group C. Control, the allantoic fluid-containing virus; group A, PAW interacted with virus suspension at a volume ratio of 9:1 (PAW: virus = 9:1); group B, PAW interacted with virus suspension at a volume ratio of 4:1 (PAW: virus = 4:1); group C, PAW interacted with virus suspension at a volume ratio of 2:1 (PAW: virus = 2:1)

The ORP and pH value were plotted as shown in Fig. 5(a). After PAW and allantoic fluid-containing virus were at a ratio of 9:1, 4:1, and 2:1, the pH value decreased from the initial 8.4 to 3.9, 4.1, and 4.3, respectively (open triangular symbol). There was a significant difference between the control group and PAW treatment groups. Meanwhile, it was found that pH value was linked to the treatment ratio, which decreased with the ratio increased.

ORP value of allantoic fluid was only 76.3 mV, while the ORP value increased significantly for group A, group B, and group C, reaching 558.7 mV, 485.3 mV, and 435.7 mV, respectively (filled square symbol), which showed a positive correlation with the volume ratio between PAW and NDV.

ESR was employed to measure the typical free radical NO in PAW. In Fig. 5(b), the characteristic peak of NO radical was not observed in allantoic fluid. However, the signal intensities of NO radical in group A, group B, and group C were detected obviously.

## Discussion

Animal infectious diseases, especially virus diseases, are a worldwide concern as they usually cause great loss in domestic animal and poultry industry (Alexander 1995; Ganar et al. 2014). Therefore, vaccine plays an important role in controlling and preventing virus transmission. The inactivated vaccine as widely used vaccine was prepared by some inactivating agents, such as formaldehyde and β-propiolactone, in livestock industry (Daszak et al. 2000). Considering the more serious RSV infection caused by formaldehyde-inactivated RSV vaccine and the allergic reactions caused by BPL, developing a potential method of inactivating virus and preparing vaccine is still required. In consideration of the inactivation ability of PAW on varieties of pathogens, the potential application of PAW toward inactivated vaccine preparation was investigated in this study.

In order to prepare the inactivated vaccine, the inactivation efficacy of PAW was first verified by ELA and HA assay (Fig. S1). The results demonstrated that NDV treated by PAW at a volume ratio of 9:1, 4:1, and 2:1 lost the ability to reproduce in the chicken embryo, laying a foundation for the vaccine preparation. The results implied that the operation of inactivating virus while maintaining its immunogenicity was correlated with treatment ratio. Virus still maintained its viability when the treatment ratio was 1:1 (data not shown), which was consistent with the above results.

In combination with the results of HI and ELISA, it was found that chicken injected with group B displayed positive HI titers and S/P ratios, inducing comparable antibody levels to formaldehyde-inactivated vaccine, which proved that group B effectively enhanced humoral-mediated immune responses.

In order to investigate immunogenic potential of the PAW-inactivated vaccine for stimulating immune responses in SPF chickens, the lymphocyte proliferation was assessed, which was the indicator reflecting the cellular immunity state in the animal (Huang et al. 2008). The results demonstrated that formaldehyde-inactivated vaccine and group B significantly improved the lymphocyte proliferation, indicating that group B might have the potential to elicit cellular-mediated immune responses.

Cell-mediated immunity played an integral role in the development of protection in NDV vaccine immunized chickens and involved in viral clearance (Kapczynski et al. 2013). In this study, the distribution of CD3^+^ T cells and their subsets CD3^+^ CD4^+^ cells and CD3^+^ CD4^+^ were assessed. The flow cytometry analysis showed significantly higher levels of CD3^+^ and CD4^+^ cells in group B compared with that in group A and group C (Table 1), which were in accordance with HI and ELISA results. These data suggested that group B induced stronger immune response at the cellular level than other groups. Increased cell-mediated immune responses to NDV were also reported by many reports (Amanna et al. 2012; Epstein et al. 2011; Pinto et al. 2013). It has been realized that CD4^+^ cells arising during both chronic and acute infection were pivotal against pathogens that evade the classical class I processing pathway (Brown et al. 2009; Jellison et al. 2005). The results indicated that PAW-inactivated vaccine increased more CD4^+^ T cell populations compared with CD8^+^ T cell populations. Previous research has also reported that vaccination with formalin-inactivated respiratory syncytial virus had an increased number of CD4^+^ cells but a decreased number of CD8^+^ cells (Waris et al. 1996). Nevertheless, the role of cellular immune responses and cytokines like gamma interferon (IFN-γ), interleukin-18 (IL-18), and IL-4 in the immunization efficacy of PAW-inactivated vaccine still needs further studies.

Strong humoral immunity and cellular immunity was the critical factor that animal resists diseases, especially infectious diseases. The increasing counts of CD3^+^ T cells and CD4^+^ T cells will enhance cell-mediated immunity. The results indicated that group B could significantly promote the activation potential of T cells in chicken. The challenge assay further confirmed that NDV-specific immune response caused by group B provided significant protection (Fig. 4) while group A and group C not protective.

To illuminate the possible reaction mechanism of PAW and virus solution, the physicochemical properties of PAW were evaluated. ORP value represented the overall oxidizing ability of the solution, which was involved as a pivotal role in the virus inactivation (Kim et al. 2010b; Zhang et al. 2013). The augment in ORP value indicated that the antioxidant capacity of solution increased remarkably, which have a close relationship with the treatment ratio. Previous research has proved that high ORP and low pH were the main causes of non-thermal plasma antiviral effects. It was speculated that a series of chemical reactions occurred in the system of PAW and allantoic fluid system. It has been realized that varieties of ROS in cold plasma could destroy the viral envelope protein and damage the nucleic acid, thus leading to virus inactivation (Guo et al. 2018; Morita et al. 2000; Su et al. 2018; Tagawa et al. 2000; Wu et al. 2015; Xia et al. 2019), which was in a good agreement with our results.

NO radical was detected by ESR, which was an active species and easily transformed to other nitrogenous compounds (Laroussi 2005). There was no significant difference among group A, group B, and group C, which was conjectured that other radicals existed in PAW contributed to ORP values. NO radical reacted with the protein and other substances, causing many biological reactions and disturbing normal viral replication. The decrease in infectivity was closely related to the breakage of protein and RNA of the virus (Tamaki et al. 2014). We speculated that RONS in the system led to a significant increase in the ORP values, which resulted in inactivation of the virus.

Group A and group C elicited lower NDV-specific antibody titers than group B, meaning that the antibody level was dependent on treatment ratio to some extent. In light of the analyses above, we conjectured that excessive PAW volumes might damage the immunogenicity of virus, contributing to the lower production of antibody titers. On the other hand, PAW probably possessed the function of immunoadjuvant. Moderate PAW treatment induced strong humoral response, which may explain why the antibody levels elicited by group C were lower than that by group B. Overall, the immune response induced by PAW-inactivated vaccine was dose-dependent, including NDV-specific antibody production and a percentage of specific CD4^+^ T cells.

It is important to investigate how strong the oxidation ability affects pivotal factors contributing to virus inactivation and immunogenicity retention. The long-lived and short-lived radicals in PAW decayed over time. Thus, it was considered as an environmental friendly solution. Based on this, PAW was expected as an alternative technology to control devastating infectious diseases and prepare inactivated vaccine.

In summary, PAW was used to inactivate the allantoic fluids-containing NDV for preparation of inactivated NDV vaccine. The results of SPF chicken experiments suggested that it was feasible for PAW, at an appropriate volume ratio, to prepare inactivated NDV vaccines and to induce similar specific titers of antibody compared with the conventional formaldehyde inactivation method. The flow-cytometric analysis indicated that PAW-inactivated vaccine could potentiate immune response at cellular levels and possess the protective capacity against virulent NDV in SPF chickens. In addition, RONS were believed to play a significant role during the preparation process of inactivated vaccine. As an alternative strategy, the proposed PAW technology could be suitable for use in the NDV vaccine, which lay a foundation for vaccine preparation in livestock industry.

## Electronic supplementary material

ESM 1 (PDF 790 kb)
