# Supplementary material for: Preparation of the inactivated Newcastle disease vaccine by plasma activated water and evaluation of its protection efficacy
Source: Appl Microbiol Biotechnol. 2019 Nov 16;104(1):107–17. doi: 10.1007/s00253-019-10106-8 (PMC6942578; doi:10.1007/s00253-019-10106-8)
Supplement: Supplementary file 1 — (PDF 790 kb) [file 253_2019_10106_MOESM1_ESM.pdf]

Applied Microbiology and Biotechnology

Preparation of the inactivated Newcastle disease vaccine by plasma activated water and evaluation of its protection efficacy

Hongzhuan Zhou[a,§], Ying Tian[b,§], Xia Su[a,§], Jinsong Guo[c], Zhenhua Zhang[a], Beiyu Jiang[a], Yanyan Chang[a], Lulu Lin[a], Jue Zhang[b,c,*], Bing Yang[a,*], Jing Fang[b,c]

[a] Beijing Key Laboratory for Prevention and Control of Infectious Diseases in Livestock and Poultry, Institute of Animal Husbandry and Veterinary Medicine, Beijing Academy of Agriculture and Forestry Sciences, Beijing 100097, People's Republic of China

[b] Academy for Advanced Interdisciplinary Studies, Peking University, Beijing 100871, People's Republic of China

[c] College of Engineering, Peking University, Beijing 100871, People's Republic of China

[§] Hongzhuan Zhou, Ying Tian and Xia Su contributed equally to this work.

*Co-Corresponding Author: Bing Yang, Beijing Academy of Agriculture and Forestry Sciences, Beijing, P. R. China

Email: byang111@yeah.net, Tel: +8610-51503203

*Co-Corresponding Author: Jue Zhang, Peking University, Beijing, P. R. China

Email: zhangjue@pku.edu.cn, Tel: +8610-62755036

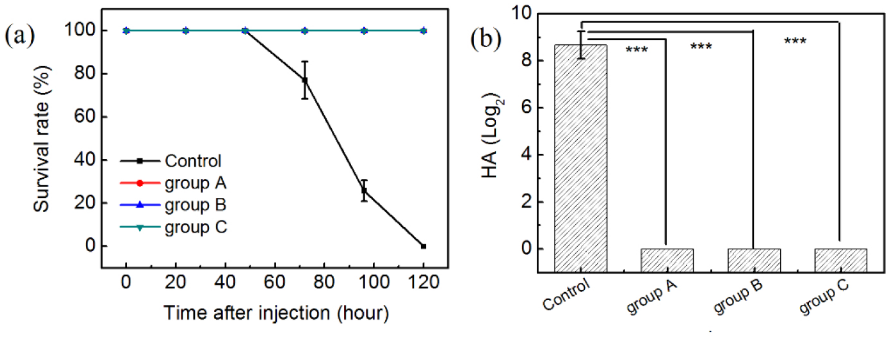

Fig. S1 Inactivation ability of PAW against NDV. (a) The survival rate of embryos during 120 h after injection of inactivated NDV treated by PAW. (b) The HA titers of allantoic fluid harvested from embryos inoculated with PAW-treated NDV. *** represents a significant difference between the PAW groups and the control group; *** $p < 0.001$. group A, PAW interacted with virus suspension at a volume ratio of 9:1 (PAW: virus = 9:1); group B, PAW interacted with virus suspension at a volume ratio of 4:1 (PAW: virus = 4:1); group C, PAW interacted with virus suspension at a volume ratio of 2:1 (PAW: virus = 2:1)
